# Quantitative Analysis of Vasodilatory Action of Quercetin on Intramural Coronary Resistance Arteries of the Rat *In Vitro*

**DOI:** 10.1371/journal.pone.0105587

**Published:** 2014-08-21

**Authors:** Anna Monori-Kiss, Emil Monos, György L. Nádasy

**Affiliations:** Institute of Human Physiology and Clinical Experimental Research, Semmelweis University, Budapest, Hungary; Osaka University Graduate School of Medicine, Japan

## Abstract

**Background:**

Dietary quercetin improves cardiovascular health, relaxes some vascular smooth muscle and has been demonstrated to serve as a substrate for the cyclooxygenase enzyme.

**Aims:**

1. To test quantitatively a potential direct vasodilatory effect on intramural coronary resistance artery segments, in different concentrations. 2. To scale vasorelaxation at different intraluminal pressure loads on such vessels of different size. 3. To test the potential role of prostanoids in vasodilatation induced by quercetin.

**Methods:**

Coronary arterioles (70–240 µm) were prepared from 24 rats and pressurized in PSS, using a pressure microangiometer.

**Results:**

The spontaneous tone that developed at 50 mmHg was relaxed by quercetin in the 10^−9^ moles/lit concentration (p<0.05), while 10^−5^ moles/lit caused full relaxation. Significant relaxation was observed at all pressure levels (10–100 mmHg) at 10^−7^ moles/lit concentration of quercetin. The cyclooxygenase blocker indomethacin (10^−5^moles/lit) induced no relaxation but contraction when physiological concentrations of quercetin were present in the tissue bath (p<0.02 with Anova), this contraction being more prominent in smaller vessels and in the higher pressure range (p<0.05, Pearson correlation). A further 2–8% contraction could be elicited by the NO blocker L-NAME (10^−4^ moles/lit).

**Conclusion:**

These results demonstrate that circulating levels of quercetin (10^−7^ moles/lit) exhibit a substantial coronary vasodilatory effect. The extent of it is commeasurable with that of several other physiological mechanisms of coronary blood flow control. At least part of this relaxation is the result of an altered balance toward the production of endogenous vasodilatory prostanoids in the coronary arteriole wall.

## Introduction

Dietary polyphenols, with quercetin being a member of this group, are present in substantial amount in various vegetables, fruits, vine and tea, and have beneficial effects on the vessel wall [1]–[3]. They delay atherosclerotic processes [4] and reduce hypertension [1], [5]–[7]. Quercetin and rutin components of food are easily absorbed, and rutin is transformed into quercetin [8]. Its glucuronide and sulfate conjugates appear in blood plasma [9]–[11]. These metabolites are cleaved in the peripheral tissues in situ, vessel wall included [12]. A vasorelaxation effect was described both in vivo and in vitro experiments [13]–[15]. While tissue blood flow is controlled dominantly by resistance arteries, quercetin effects have been studied mainly on larger vessels. Both endothelium dependent [16] and independent [17] mechanisms of quercetin-induced vasorelaxation have been described. They can be different in different vascular fields. Quercetin was found to be a reducing co-substrate of the second step in the cyclooxygenase (COX) enzymatic reaction [18], thus affecting endogenous prostanoid production [19].

Intramural coronary resistance arteries have a marked spontaneous myogenic tone which is substantially modified by endothelial relaxation and endogenous prostanoid production. Both vasoconstrictor and vasodilator prostanoids are produced by intramural coronary resistance arteries, vasoconstrictors prevailing at higher intraluminal pressures [20].

One of the aims of the present study was to test quantitatively if coronary resistance arteries do show a direct vasodilation in response to physiological concentrations of quercetin. In addition, we investigated the quercetin effect on coronary arterioles of different sizes at intact, cylindrical vascular geometry, as according to earlier observations, pharmacological modulation of smaller and larger resistance arteries may differ substantially [21]. After we have found a significant dilation effect, it was assumed that this action could be initiated at least partly through altered production of endogenous vasoactive prostanoids. This hypothesis was tested by indomethacin inhibition of COX activity in vessel segments of different caliber and at different intraluminal pressures.

## Materials and Methods

### Pressure microangiometry

Male Wistar rats, weighing 350–450 gr were anesthetized by pentobarbital (Nembutal, CEVA, 45 mg/kg body weight i.p.). All procedures conformed the Guide for the Care and Use of Laboratory Animals (NIH, 1996), the legal and institutional guidelines for animal care and were approved by the Animal Care Committee of the Semmelweis University.

The heart was removed and put in cold oxygenized physiological salt solution (PSS). A terminal branch of the left anterior descendent coronary artery (intramural small artery in the rat) was prepared from the ventricular muscle tissue. Vessel segments with no or with limited number of side branches, having outer diameter of 150–250 µm in situ were selected. Preparation was made as described earlier [22]. Excised segments, with lengths over 2.0 mm were cannulated at both ends using microcannulas (outer diameter ∼130 µm) and mounted in a glass bottomed tissue bath. Their length was extended to the original in situ value. Segments were pressurized with saline connecting them to servo-controlled pumps (Living Systems, Burlington, VT, US). The temperature of the bath was thermostated at 37°C, and bubbled with a gas mixture of 5% CO_2_, 20% O_2_ and 75% of N_2_, keeping pH at 7.4. A continuous superfusion velocity of 2.8 ml/min was ensured. The bath was positioned on the stage of an inverted microscope (Leica), the pictures of the segments were taken by a digital camera (Leica DFC 320) and an image analyzing software (Leica Qwin). Outer and inner diameters of segments were measured off-line on frozen pictures. Calibration was made using a micrometer etalon (Wild).

### Materials

All drugs, including quercetin, were purchased from Sigma-Aldrich. The composition of the PSS (Krebs-Ringer solution) we used: NaCl 119, KCl 4.7, NaH_2_PO_4_ 1.2, MgSO_4_ 1.17, NaHCO_3_ 24, CaCl_2_ 2.5, glucose 5.5, and EDTA 0.034 (in mmol/lit). The Ca^2+^-free PSS contained: NaCl 92, KCl 4.7, NaH_2_PO_4_ 1.18, MgCl_2_ 20, MgSO_4_ 1.17, NaHCO_3_ 24, glucose 5.5, EGTA 2, and EDTA 0.025 (in mmol/lit).

### *In vitro* protocols

In the first series of experiments, concentration-response curves with quercetin were taken on coronary resistance artery segments from 8 rats. To do that, segments were pressurized at 50 mmHg. This pressure was continuously kept throughout the measurement. Such segments develop substantial spontaneous tone under similar circumstances. After 40 minutes of equilibration, superfusion was changed to PSS containing increasing concentrations of quercetin from 10^−9^ to 10^−5^ moles/lit. (Physiological concentrations in humans are reported to be 10^−7^ moles/lit [7].) 20 min equilibration was left for all steps, at the end of which stable diameters were registered. Reversibility was checked by repeated administration of PSS. Finally, calcium-free PSS was superfused (20 minutes) and relaxed diameters were measured. Concentration-response curves were determined. Relaxation from spontaneous tone was expressed in percents of fully relaxed diameter.

In a second series, pressure-radius curves were taken using 16 similar vessel segments. Pressure was raised in 10 mmHg steps from 10 up to 100 mmHg. Then 10^−7^ mol/lit quercetin containing PSS solution was superfused, and the pressure-radius curves were repeatedly taken. Next, the cyclooxygenase blocker indomethacin was applied (10^−5^ moles/lit) with the quercetin still in the bath. NO effect was tested by additional application of the NO blocker L-NAME (10^−4^ moles/lit). Reproducibility was controlled by repeated measurements with 10^−7^ moles/lit quercetin alone and then with PSS without any drug added (spontaneous tone). The experiment was terminated by superfusion with Ca^2+^-free PSS (full relaxation). Contractions and relaxations were expressed in % of inner diameter measured at the same pressure in Ca^2+^-free PSS.

### Statistical procedures

Values are expressed as means ± SEM. Statistical analysis was performed using paired t-test, or two-way repeated measures ANOVA, followed by Tukey post-hoc test. Values of p<0.05 were considered statistically significant.

## Results

**Figure 1:**
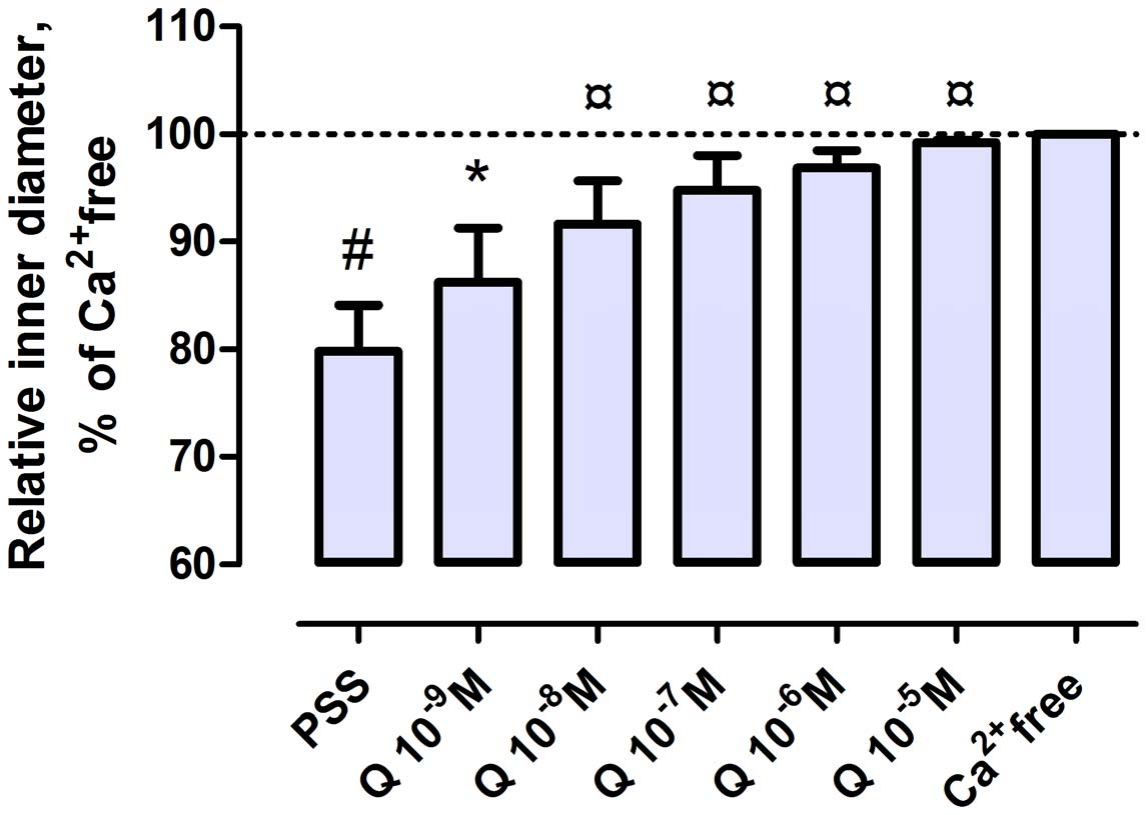
Concentration-response curve of quercetin on coronary resistance arterioles of rat. Intramural coronary arteriole segments were spontaneously contracted, then dilatated by quercetin at intraluminal pressure 50 mmHg. (PSS, Krebs-Ringer solution, Ca^2+^-free, Ca^2+^-free solution). #, significantly different from fully relaxed; ¤, different from spontaneously contracted with ANOVA; * different from spontaneously contracted with the paired t-test.

In the first series, the vessel segments incubated in oxygenized PSS exhibited a substantial spontaneous tone. At 50 mmHg intravascular pressure, the average inner diameter was 127±25 µm, which corresponds to a 20.2±4.3% contraction from fully relaxed state. Increasing concentrations of quercetin induced increasing relaxations. In 10^−9^ moles/lit concentration, statistically significant relaxation was seen, while 10^−5^ moles/lit fully relaxed the segments. This relaxation (reduction of spontaneous tone) was found to be reversible, as original tone returned upon washing off the substance (Fig. 1.).

**Figure 2:**
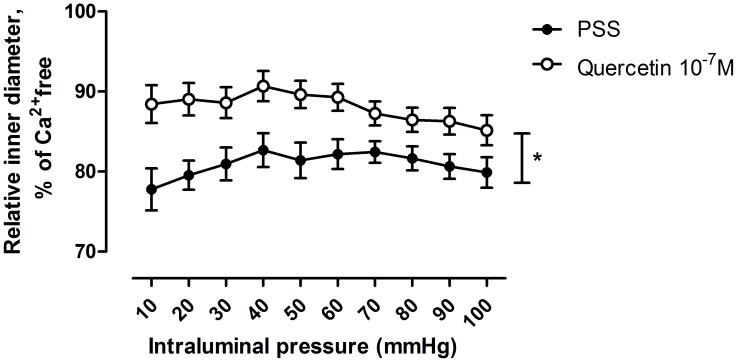
Quercetin dilation on spontaneously contracted small coronary arterioles at different pressures. Full circles represents diameters in PSS, empty circles are diameters in quercetin 10^−7^ moles/lit. * p<0.05 with ANOVA

**Figure 3:**
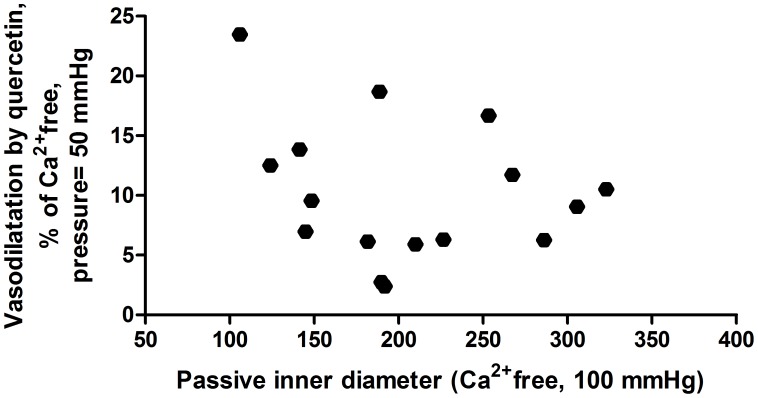
Quercetin induced dilatation as function of morphological lumen size. We found no correlation (p>0.05 with Pearson's correlation).

In the second series of experiments, 10^−7^ mol/lit quercetin concentration was further tested on pressure-radius curves in the pressure range of 10–100 mmHg. A massive dilation was found at all pressure levels studied (Fig. 2.). There was no significant correlation between the extent of quercetin-dilation and the morphological diameter of the segments (Fig. 3).

**Figure 4:**
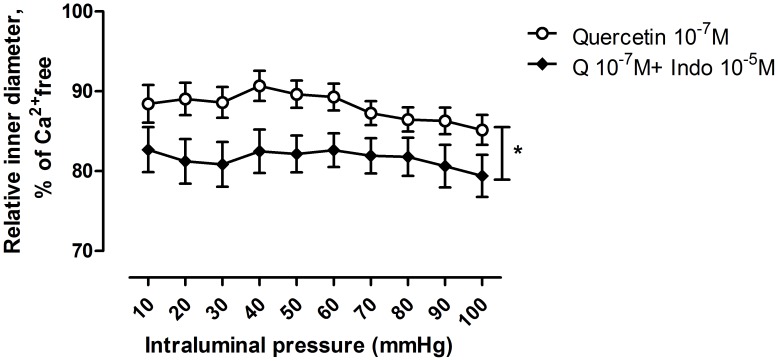
Effect of indomethacin on inner diameter of quercetin-treated coronary arteriole segments. Note contraction with indomethacin (p<0.05 with ANOVA), in contrast the expected vasodilation, indicating the presence of vasodilatory prostanoids with the polyphenol. Indomethacin concentration was 10^−5^ moles/lit, represented by full circles, quercetin was in 10^−7^ moles/lit concentration, represented by empty circles.

**Figure 5:**
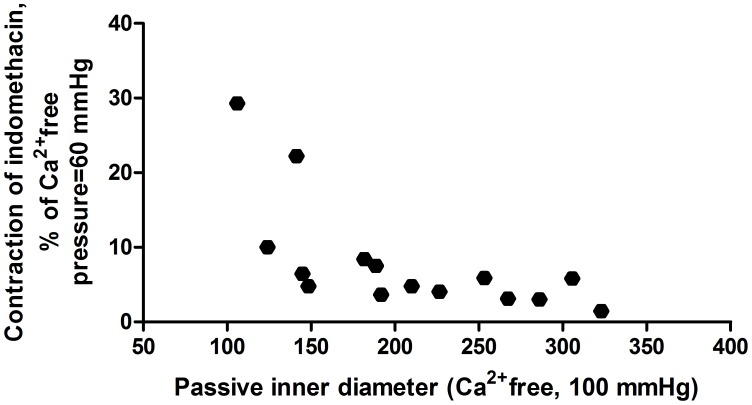
Correlation of indomethacin induced contraction on quercetin-dilated segments with the morphological lumen size. Note a more prominent contraction in smaller vessels (p<0.05 with Pearson's correlation).

The COX blocker indomethacin, in a concentration of 10^−5^ moles/lit induced a significant contraction of all quercetin treated segments at all pressure levels (Fig. 4.). This effect seems to depend on morphological lumen size: a statistically significant negative correlation between morphological diameter (Ca^2+^-free solution, 100 mmHg pressure) and indomethacin induced contraction at higher pressures (60–100 mmHg) was found (Fig. 5.).

**Figure 6:**
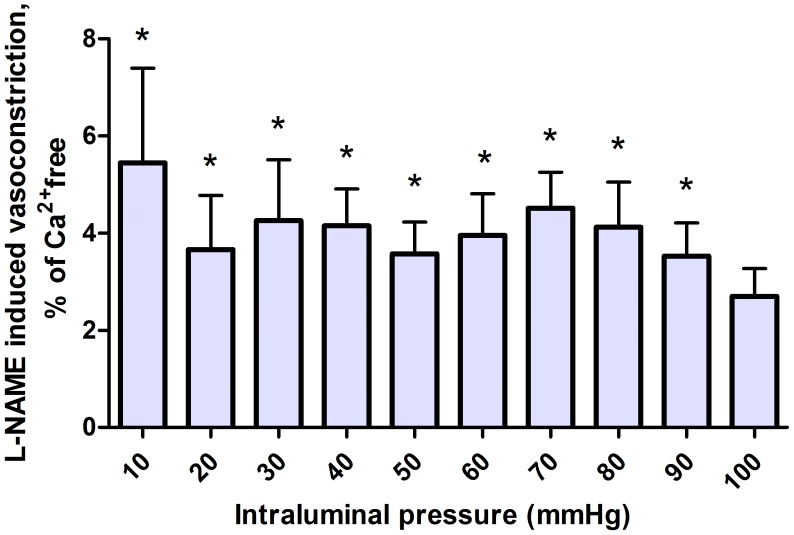
Vasoconstriction induced by L-NAME in coronary arteriole segments pretreated with quercetin and indomethacin. Concentrations: L-NAME: 10^−4^ moles/lit, quercetin: 10^−7^ moles/lit, and indomethacin: 10^−5^ moles/lit. * p<0.05 with ANOVA

Finally, the NO-blocker L-NAME was given (10^−4^ moles/lit) to test the level of endothelial vasodilation. Significant contraction was found, but its extent varied only between 2–8% of relaxed diameter (Fig. 6.).

## Discussion

In this study it was proved that the polyphenol quercetin elicits a direct concentration-dependent vasodilation on intramural coronary resistance arteries at physiologically realistic concentrations. In the very low concentration of 10^−9^ moles/lit a slight but significant relaxation was found in the intrinsic spontaneous tone. Equivalent to the physiological concentration range of 10^−7^ moles/lit in humans [7] resulted in 10.1±1.5% elevation in the inner diameter. Close to the therapeutic concentrations of 10^−6^ moles/lit quercetin [7], the spontaneous vascular tone almost fully diminished. Quercetin, it proves, can be listed among potent known coronary vasodilators. A direct vasodilatory action of quercetin has been demonstrated until now exclusively on ring preparations of larger vessels, on major coronary arteries [13]–[17], [23], [24], as well as on resistance size mesenteric [17], and coronary arteries [25]. The pressure microangiometry method we applied was able to demonstrate vasodilatory actions of quercetin at physiologically and pharmacologically realistic concentrations, at real intraluminal pressures and inducing hemodynamically substantial alterations in inner diameter.

In addition, we found, that relaxation caused by 10^−7^ moles/lit quercetin on the small coronary arterioles does not depend on vessel diameter in the range of 70–250 µm, all samples relaxed by appr.10 percents. It means that the spontaneous tone was reduced to its half in each segment simply by the presence of physiological concentrations of quercetin in the tissue bath. As smaller coronary resistance arteries have larger tone, the vasorelaxation induced on smaller vessels were more potent. Considering Hagen-Poiseuille's law, this might induce approximately 22–28% increase in flow at larger arterioles (over 150 µm diameter), and 42–50% increase at smaller coronaries (under 150 µm diameter).

Our measurements provide some further insight into the possible mechanism of this vasodilatory action. Quercetin treated coronary arteriole segments contracted at all transmural pressures studied in response to the cyclooxygenase (COX) blocker indomethacin, proving the presence and activity of vasodilatory endogenous prostanoids in the vascular wall under the polyphenol effect. According to earlier observations, without quercetin, similar preparations produce mostly vasoconstrictor prostanoids when challenged with increasing intraluminal pressures [26]. This points to one potential mechanism of the vasodilation: altered balance toward vasodilatory prostanoids in the coronary resistance artery wall. As to the molecular mechanism, quercetin was reported to be a modulator in the COX enzymatic reaction [19], in this way affecting the endogenous prostanoid production [26], [27]. Our results are in good agreement with these observations. Pharmacological modulation of vasoactive endogenous prostanoid production in the vessel wall has been demonstrated earlier with our contribution [26], [27].

Addition of the NO blocker L-NAME to the tissue bath induced vasoconstriction, but its extent (2–8% at different pressures) does not indicate a substantial NO-dependent vasorelaxing effect of quercetin. Namely, it is not larger than the L-NAA induced contractions of similar segments with indomethacin in the bath without the presence of quercetin [26]. It is well-known that quercetin exhibits antioxidant [28], [29] and ROS scavenging properties [30]. This can help to preserve NO, and provide longer half-life time, to express vasodilatation [31].

In conclusion, these experiments suggest that physiological circulating levels of quercetin continuously keep activated a coronary vasodilatory system, of which significance and extent is comparable with those of several other physiological mechanisms of coronary resistance control. Our observations suggest that production of endogenous vasodilatory prostanoids depends on the presence of such reducing agents as the polyphenols. Here, we can cite the original view of the Nobel-prize winner Hungarian biochemist Albert Szentgyörgyi, who suggested to recognize dietary polyphenols as vitamins [32] (“vitamin P” he called them). This view was challenged later because of absence of manifest deficiency symptoms [33]. We assume that the prevailing massive metabolic control (hypoxic vasodilation) of cardiac tissue perfusion easily overrides the missing polyphenol-relaxation, this way masking deficiency symptoms. However, if under pathological circumstances, the balance of vasoconstriction-relaxation is disturbed, it might reduce remaining control capacity. Maintaining a physiological quercetin concentration in the blood plasma may help correct the balance toward vasorelaxation in coronary arterioles.

## References

[1] Pechanova O, Bernatova I, Babal P, Martinez MC, Kysela S, et al (2004) Red wine polyphenols prevent cardiovascular alterations in L-NAME-induced hypertension. J Hypertens 22: 1551–1559. 15257179 10.1097/01.hjh.0000133734.32125.c7

[2] Kurin E, Atanasov AG, Donath O, Heiss EH, Dirsch VM, et al (2012) Synergy study of the inhibitory potential of red wine polyphenols on vascular smooth muscle cell proliferation. Planta Med 78: 772–778. 22499559 10.1055/s-0031-1298440

[3] Scoditti E, Calabriso N, Massaro M, Pellegrino M, Storelli C, et al (2012) Mediterranean diet polyphenols reduce inflammatory angiogenesis through MMP-9 and COX-2 inhibition in human vascular endothelial cells: a potentially protective mechanism in atherosclerotic vascular disease and cancer. Arch Biochem Biophys 527: 81–89. 22595400 10.1016/j.abb.2012.05.003

[4] Yao LH, Jiang YM, Shi J, Tomas-Barberan FA, Datta N, et al (2004) Flavonoids in food and their health benefits. Plant Foods Hum Nutr 59: 113–122. 15678717 10.1007/s11130-004-0049-7

[5] Sarr M, Chataigneau M, Martins S, Schott C, El Bedoui J, et al (2006) Red wine polyphenols prevent angiotensin II-induced hypertension and endothelial dysfunction in rats: role of NADPH oxidase. Cardiovasc Res 71: 794–802. 16822492 10.1016/j.cardiores.2006.05.022

[6] Medina-Remon A, Zamora-Ros R, Rotches-Ribalta M, Andres-Lacueva C, Martinez-Gonzalez MA, et al (2011) Total polyphenol excretion and blood pressure in subjects at high cardiovascular risk. Nutr Metab Cardiovasc Dis 21: 323–331. 20167460 10.1016/j.numecd.2009.10.019

[7] Edwards RL, Lyon T, Litwin SE, Rabovsky A, Symons JD, et al (2007) Quercetin reduces blood pressure in hypertensive subjects. J Nutr 137: 2405–2411. 17951477 10.1093/jn/137.11.2405

[8] Kim DH, Jung EA, Sohng IS, Han JA, Kim TH, et al (1998) Intestinal bacterial metabolism of flavonoids and its relation to some biological activities. Arch Pharm Res 21: 17–23. 9875509 10.1007/BF03216747

[9] Manach C, Williamson G, Morand C, Scalbert A, Remesy C (2005) Bioavailability and bioefficacy of polyphenols in humans. I. Review of 97 bioavailability studies. Am J Clin Nutr 81: 230s–242s. 15640486 10.1093/ajcn/81.1.230S

[10] Manach C, Morand C, Texier O, Favier ML, Agullo G, et al (1995) Quercetin metabolites in plasma of rats fed diets containing rutin or quercetin. J Nutr 125: 1911–1922. 7616308 10.1093/jn/125.7.1911

[11] Mullen W, Boitier A, Stewart AJ, Crozier A (2004) Flavonoid metabolites in human plasma and urine after the consumption of red onions: analysis by liquid chromatography with photodiode array and full scan tandem mass spectrometric detection. J Chromatogr A 1058: 163–168. 15595664

[12] Perez-Vizcaino F, Duarte J, Santos-Buelga C (2012) The flavonoid paradox: conjugation and deconjugation as key steps for the biological activity of flavonoids. J Sci Food Agric 92: 1822–1825. 22555950 10.1002/jsfa.5697

[13] Rendig SV, Symons JD, Longhurst JC, Amsterdam EA (1998) Quercetin, a biologically active flavonoid, relaxes isolated rabbit coronary arteries. Faseb J 12: A405–A405.

[14] Ibarra M, Perez-Vizcaino F, Cogolludo A, Duarte J, Zaragoza-Arnaez F, et al (2002) Cardiovascular effects of isorhamnetin and quercetin in isolated rat and porcine vascular smooth muscle and isolated rat atria. Planta Med 68: 307–310. 11988852 10.1055/s-2002-26752

[15] Fusi F, Saponara S, Pessina F, Gorelli B, Sgaragli G (2003) Effects of quercetin and rutin on vascular preparations: a comparison between mechanical and electrophysiological phenomena. Eur J Nutr 42: 10–17. 12594537 10.1007/s00394-003-0395-5

[16] Taubert D, Berkels R, Klaus W, Roesen R (2002) Nitric oxide formation and corresponding relaxation of porcine coronary arteries induced by plant phenols: essential structural features. J Cardiovasc Pharmacol 40: 701–713. 12409979 10.1097/00005344-200211000-00008

[17] Perez-Vizcaino F, Ibarra M, Cogolludo AL, Duarte J, Zaragoza-Arnaez F, et al (2002) Endothelium-independent vasodilator effects of the flavonoid quercetin and its methylated metabolites in rat conductance and resistance arteries. J Pharmacol Exp Ther 302: 66–72. 12065701 10.1124/jpet.302.1.66

[18] Wang P, Bai H-W, Zhu BT (2010) Structural Basis for Certain Naturally Occurring Bioflavonoids to Function as Reducing Co-Substrates of Cyclooxygenase I and II. PLoS ONE 5: e12316. 20808785 10.1371/journal.pone.0012316 PMC2925883

[19] Al-Fayez M, Cai H, Tunstall R, Steward WP, Gescher AJ (2006) Differential modulation of cyclooxygenase-mediated prostaglandin production by the putative cancer chemopreventive flavonoids tricin, apigenin and quercetin. Cancer Chemother Pharmacol 58: 816–825. 16552572 10.1007/s00280-006-0228-3

[20] Szekeres M, Kaley G, Nadasy GL, Dezsi L, Koller A (2006) Nitric oxide modulates the interaction of pressure-induced wall mechanics and myogenic response of rat intramural coronary arterioles. Acta Physiol Hung 93: 1–12. 16830688 10.1556/APhysiol.93.2006.1.1

[21] Szekeres M, Dezsi L, Nadasy GL, Kaley G, Koller A (2001) Pharmacologic inhomogeneity between the reactivity of intramural coronary arteries and arterioles. J Cardiovasc Pharmacol 38: 584–592. 11588529 10.1097/00005344-200110000-00011

[22] Nadasy GL, Szekeres M, Dezsi L, Varbiro S, Szekacs B, et al (2001) Preparation of intramural small coronary artery and arteriole segments and resistance artery networks from the rat heart for microarteriography and for in situ perfusion video mapping. Microvasc Res 61: 282–286. 11336539 10.1006/mvre.2000.2297

[23] Xu YC, Leung SW, Yeung DK, Hu LH, Chen GH, et al (2007) Structure-activity relationships of flavonoids for vascular relaxation in porcine coronary artery. Phytochemistry 68: 1179–1188. 17395220 10.1016/j.phytochem.2007.02.013

[24] Suri S, Liu XH, Rayment S, Hughes DA, Kroon PA, et al (2010) Quercetin and its major metabolites selectively modulate cyclic GMP-dependent relaxations and associated tolerance in pig isolated coronary artery. Br J Pharmacol 159: 566–575. 20050852 10.1111/j.1476-5381.2009.00556.x PMC2828021

[25] Rendig SV, Symons JD, Longhurst JC, Amsterdam EA (2001) Effects of red wine, alcohol, and quercetin on coronary resistance and conductance arteries. J Cardiovasc Pharmacol 38: 219–227. 11483871 10.1097/00005344-200108000-00007

[26] Szekeres M, Nadasy GL, Kaley G, Koller A (2004) Nitric oxide and prostaglandins modulate pressure-induced myogenic responses of intramural coronary arterioles. J Cardiovasc Pharmacol 43: 242–249. 14716212 10.1097/00005344-200402000-00012

[27] Szekacs B, Nadasy GL, Vajo Z, Juhasz I, Feher J, et al (1996) Prostacyclin and thromboxane production of rat and cat arterial tissue is altered independently by several vasoactive substances. Prostaglandins 52: 221–235. 8908622 10.1016/s0090-6980(96)00099-8

[28] Molina MF, Sanchez-Reus I, Iglesias I, Benedi J (2003) Quercetin, a flavonoid antioxidant, prevents and protects against ethanol-induced oxidative stress in mouse liver. Biol Pharm Bull 26: 1398–1402. 14519943 10.1248/bpb.26.1398

[29] Maciel RM, Costa MM, Martins DB, Franca RT, Schmatz R, et al (2013) Antioxidant and anti-inflammatory effects of quercetin in functional and morphological alterations in streptozotocin-induced diabetic rats. Res Vet Sci 95: 389–397. 23706762 10.1016/j.rvsc.2013.04.028

[30] Rezaei-Sadabady R, Eidi A, Zarghami N, Barzegar A (2014) Intracellular ROS protection efficiency and free radical-scavenging activity of quercetin and quercetin-encapsulated liposomes. Artif Cells Nanomed Biotechnol: 1–7. 10.3109/21691401.2014.926456 24959911

[31] Machha A, Achike FI, Mustafa AM, Mustafa MR (2007) Quercetin, a flavonoid antioxidant, modulates endothelium-derived nitric oxide bioavailability in diabetic rat aortas. Nitric Oxide 16: 442–447. 17513143 10.1016/j.niox.2007.04.001

[32] Rusznyák ST, Szent-Györgyi A (1936) Vitamin P: Flavonols as Vitamins. Nature 138: 27–27.

[33] Vickery HB, Nelson EM, Almquist HJ, Elvehjem CA (1950) Term "vitamin P" recommended to be discontinued. Science 112: 628. 10.1126/science.112.2917.628 14787488

